# Molecular Epidemiology and Evolution of Human Respiratory Syncytial Virus and Human Metapneumovirus

**DOI:** 10.1371/journal.pone.0017427

**Published:** 2011-03-01

**Authors:** Eleanor R. Gaunt, Rogier R. Jansen, Yong Poovorawan, Kate E. Templeton, Geoffrey L. Toms, Peter Simmonds

**Affiliations:** 1 Centre for Infectious Diseases, University of Edinburgh, Edinburgh, United Kingdom; 2 Department Medical Microbiology, Academic Medical Centre, Amsterdam, Netherlands; 3 Center of Excellence in Clinical Virology, Chulalongkorn University, Bangkok, Thailand; 4 Institute of Cellular Medicine, Newcastle University, Newcastle upon Tyne, United Kingdom; 5 Specialist Virology Centre, Royal Infirmary of Edinburgh, Edinburgh, United Kingdom; Erasmus Medical Center, Netherlands

## Abstract

Human respiratory syncytial virus (HRSV) and human metapneumovirus (HMPV) are ubiquitous respiratory pathogens of the *Pneumovirinae* subfamily of the *Paramyxoviridae*. Two major surface antigens are expressed by both viruses; the highly conserved fusion (F) protein, and the extremely diverse attachment (G) glycoprotein. Both viruses comprise two genetic groups, A and B. Circulation frequencies of the two genetic groups fluctuate for both viruses, giving rise to frequently observed switching of the predominantly circulating group. Nucleotide sequence data for the F and G gene regions of HRSV and HMPV variants from the UK, the Netherlands, Bangkok and data available from Genbank were used to identify clades of both viruses. Several contemporary circulating clades of HRSV and HMPV were identified by phylogenetic reconstructions. The molecular epidemiology and evolutionary dynamics of clades were modelled in parallel. Times of origin were determined and positively selected sites were identified. Sustained circulation of contemporary clades of both viruses for decades and their global dissemination demonstrated that switching of the predominant genetic group did not arise through the emergence of novel lineages each respiratory season, but through the fluctuating circulation frequencies of pre-existing lineages which undergo proliferative and eclipse phases. An abundance of sites were identified as positively selected within the G protein but not the F protein of both viruses. For HRSV, these were discordant with previously identified residues under selection, suggesting the virus can evade immune responses by generating diversity at multiple sites within linear epitopes. For both viruses, different sites were identified as positively selected between genetic groups.

## Introduction

Human respiratory syncytial virus (HRSV) and human metapneumovirus (HMPV) are globally ubiquitous respiratory pathogens of the *Pneumovirinae* subfamily of the *Paramyxoviridae*. Both viruses comprise two genetic groups, A and B, distinguishable genetically and serologically [1]–[3] which co-circulate with fluctuating frequencies. The two HRSV genetic groups are referred to as subgroups; these comprise genotypes distinguished on the basis of antibody cross reactivity [4] or phylogeny [5]. Each of the two HMPV genetic groups are referred to somewhat paradoxically as genotypes, and each genotype comprises two sub-genotypes (A1, A2, B1 and B2). HMPV genotypes are distinguishable serologically and sub-genotypes are discerned phylogenetically [3].

Fluctuating circulation frequencies of HRSV subtypes and HMPV genotypes give rise to the observation of switching of the predominantly circulating subtype (HRSV) or genotype (HMPV) between respiratory seasons [6]–[13]. HMPV was discovered in 2001 and so longitudinal epidemiologic studies are infrequent, though for HRSV a theme of cyclicity whereby subtype A predominates for a number of seasons then subtype B predominates (usually for a shorter duration) are reported [14]–[19]. HRSV-A is considered the major subtype in terms of both frequency [20] and associated morbidity [7]. Similarly, HMPV-A strains are generally detected at a higher frequency than HMPV-B strains [12], [13], [21] and clinical differences are reported between HMPV genotypes [21], [22]. Repeat HRSV infections occur throughout life with decreasing morbidity, and increasingly evidence suggests the same is also true for HMPV [3], [23]–[27].

The HRSV and HMPV virions both express two highly immunogenic surface proteins against which adaptive immune responses are directed. The fusion (F) protein mediates fusion of viral and cell membranes and is highly conserved. Anti-HRSV antibody directed against F protein is cross-reactive for strains of both subtypes [1], [2], [28], [29], and studies on HMPV using human sera and animal models have indicated similar antibody reactivity patterns [30]–[33]. It is conceivable that the conformational changes arising on activation of the fusion protein [34] serve to expose the conserved functional (and immunogenic) regions (analogous to the gp41 fusion protein of HIV-1 [35]) which are otherwise, in the native state, sheltered from immunologic recognition.

The attachment (G) glycoprotein of pneumoviruses conversely portrays several immune evasive traits. Specificity of antibody raised against the G protein extends to, and possibly beyond, the genotype level (HRSV) or sub-genotype level (HMPV) [4], [29], [30], [36]–[38]. In both viruses the G protein is extensively glycosylated with both *N*- and *O*-linked sugars and a high proportion of proline residues [39], [40] thought to reduce ordered secondary structure of the protein [41].

The highly variable G protein of HRSV comprises 298 [42] or 318 [43] residues. Two mucin-like hypervariable regions (HVRs) at the C terminus under significant positive selection form hydrophilic stalk like protrusions from the surface of the virion separated by an exposed [44] but conserved and non-glycosylated region comprising residues 151–190 [45]. A heparin binding domain (HBD) identified between residues 184–198 (subgroup A) or 183–197 (subgroup B) binds heparin-like glycosaminoglycans (GAG) on the host cell surface [46]. A fourteen residue region incorporating four universally encoded cysteine residues at positions 173, 176, 182 and 186 believed to form disulphide bridges is common to human and bovine RSVs [47], and the G protein has been shown to bind host cell receptor CX3CR1 via the CX3C chemokine domain accommodating cysteine residues at positions 182 and 186 [48]. Interestingly, subtype specific seroconversion directed against this antigenic region is detectable in only 40% of individuals [49].

The HMPV G protein, which comprises 217 to 236 residues [50], [51] has not been resolved in such detail. Nevertheless, great variability in the C terminal ectodomain is seen; conversely to HRSV, no conserved cysteine pairs or chemokine domains are detectable. HMPV does not encode a conserved methionine or alternative initiation codon in or adjacent to the transmembrane region which would permit production of a secreted form of the G protein, unlike HRSV [52].

It is hypothesized that switching of the predominant circulating subtype of HRSV is brought about by short-lived subtype-specific herd immunity in a population generated over one or two seasons, which favours dissemination of the alternate subtype in a subsequent season [43], [53], [54]. This suggestion has been borrowed to explain HMPV genotype switching also [12].

Evolutionary modelling of several respiratory viruses has been undertaken. A well conducted analysis which identified positively selected sites and the evolutionary characteristics of HRSV was bipartite, corresponding with the two subtypes A and B [10], [11]. Sequence data spanning 47 and 45 years (for subtypes A and B respectively) were used to identify positively selected sites within the attachment (G) protein, to distinguish residues which had a significant likelihood of being *O*-glycosylated (a mechanism used to shelter residues from immunologic recognition [55]), and to determine the time since the most recent common ancestor (*t_MRCA)_* of the HRSV species – which was estimated to have existed 350 years ago.

The evolutionary dynamics of the closely related HMPV have also been explored [56]–[58]. The *t_MRCA_* of the HMPV sub-genotypes were estimated at 12–28 years, and the two genotypes were found to have mean *t_MRCA_*s of 26–51 years [56]–[58]. The *t_MRCA_* across the species in one study was 119–133 years [56], whereas another study using sequence data collected over a greater number of years determined a more recent species level *t_MRCA_* of 97 years [58], though both analyses were conducted using similar sequence data and the same software package (BEAST [59]).

Many human infecting viruses, both in the field of respiratory medicine and more widely, undergo a turnover and replacement of predominant lineages with emergent strains. For example, clades of echoviruses 9, 11 and 30 are frequently replaced by novel recombinant forms with striking periodicity [60], dengue virus serotypes are comprised of clades undergoing replacement [61], and measles virus clade replacement is described, the latter of which is likely due to selective advantages brought about by the vaccine era [62]. HRSV and HMPV have not previously been discussed in such terms, and so it was decided to investigate whether similar evolutionary mechanisms are evident for HRSV and HMPV.

We report identification and evolutionary modelling of three geographically dispersed contemporary clades of HRSV and five of HMPV, which have circulated for decades. Switching of the predominantly circulating genotype (HMPV)/subtype (HRSV) therefore cannot be attributed to the emergence of novel virus lineages. Identification of numerous sites under positive selection in the G proteins of both viruses were frequently discordant with those identified in previous studies, and little overlap in positively selected sites was observed between HRSV subtypes or HMPV genotypes. The interpretations of these findings are discussed.

## Results

### Circulating clades of HRSV and phylogenetic reconstructions

**Figure 1:**
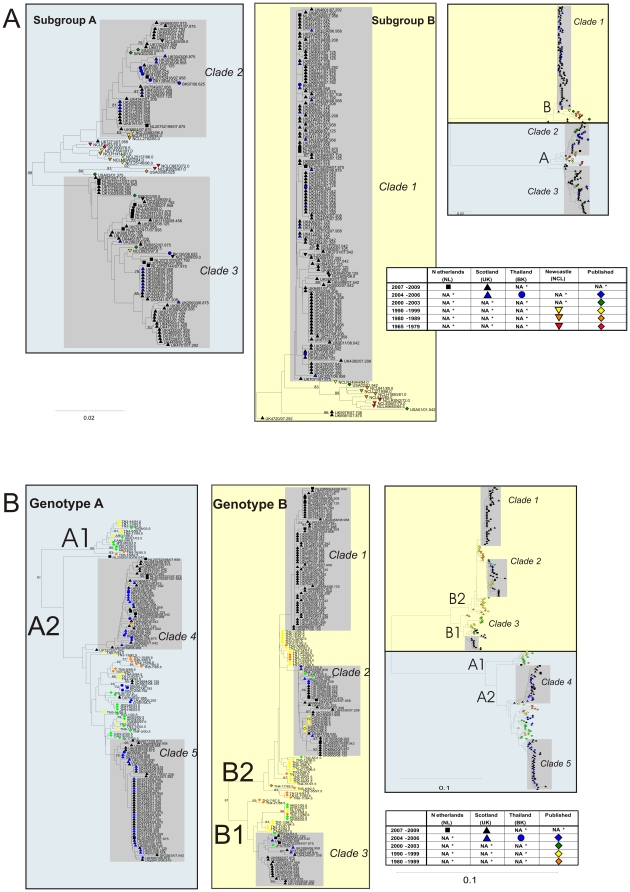
Phylogenetic analysis of HRSV (A) and HMPV (B) partial F gene sequences. Phyogenetic reconstruction was by neighbour joining of MCL-corrected pair-wise distances. Clades identified as described in the methods are indicated by grey shaded boxes. Sequence symbols are colour coded by year of isolation. Symbol shape denotes geographic origins sequences. Bootstrap values >70% are indicated. (A) Phylogenies rooted with bovine RSV (not shown). Subgroups A and B are indicated by the blue and yellow boxes respectively. (B) rooted with avian metapneumovirus species C (not shown). Genotypes A and B are indicated by the blue and yellow boxes respectively. NA, None analysed.

To investigate the geographical and temporal distribution of individual clades of each virus, phylogenetic analyses were performed on 243 HRSV and 310 HMPV F gene sequences with isolation dates spanning 44 and 26 years respectively (Fig. 1). Three clades of HRSV with sufficient sequences available were identified (labelled 1–3; Fig. 1A), of which two were subtype A, and one was subtype B.

HRSV subtype B sequences largely grouped into the one clade, and all but three subtype B sequences falling outside this clade were collected before 2002. HRSV subtype A sequences mostly fell into one of two clades, with all but one of the sequences not belonging to one of the identified clades having a collection date prior to 2002. There was little evidence of geographical clustering of HRSV sequences, with strains from Newcastle, the Netherlands and Bangkok phylogenetically interspersed among the Edinburgh strains in all three clades (Fig. 1A). HRSV clade 1 was comprised entirely of sequences generated during this study, whereas clades 2 and 3 incorporated sequences downloaded from Genbank of Asian origin.

**Figure 2:**
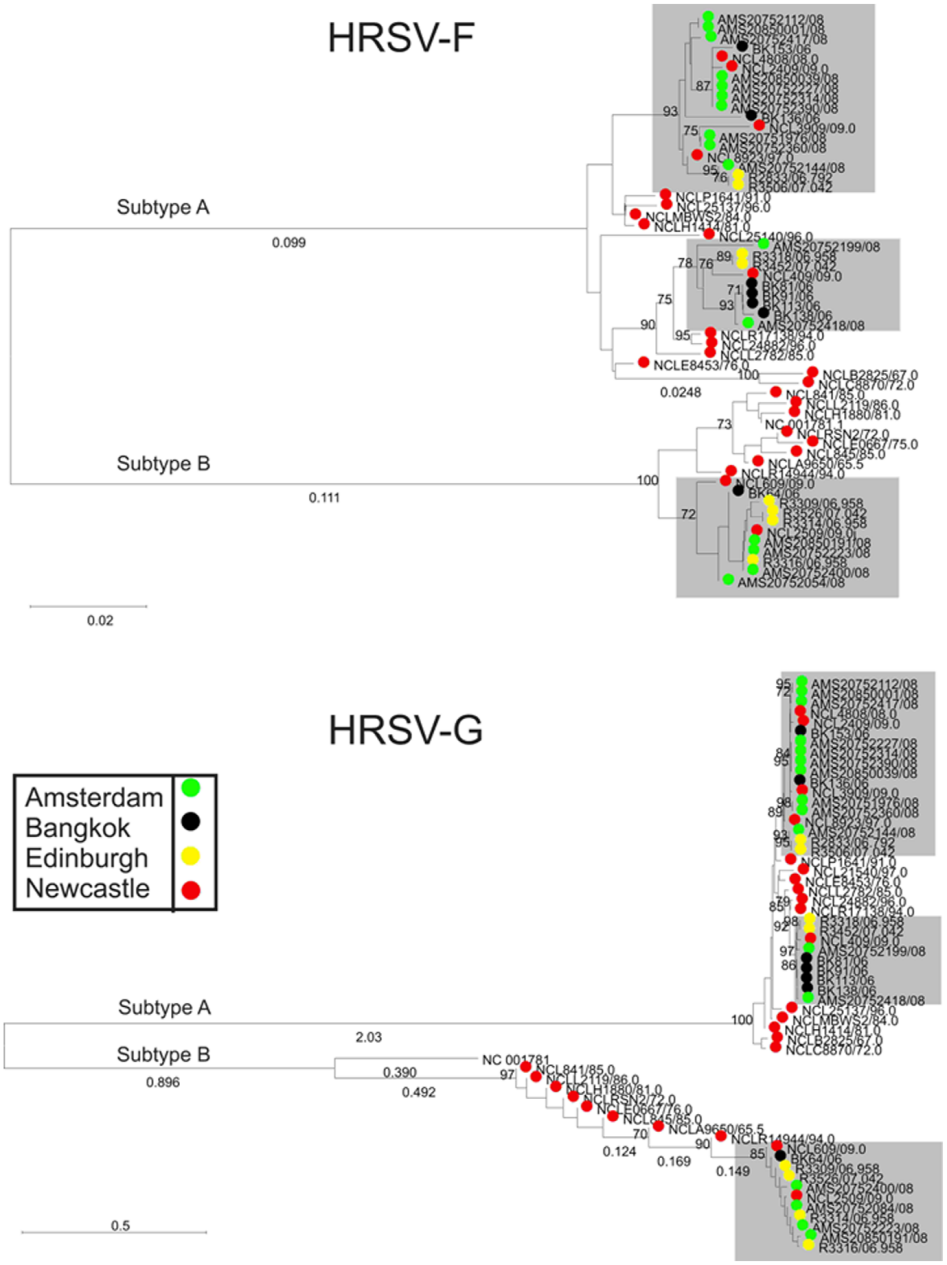
Phylogenetic analysis of 58 HRSV sequences in the F and G genes. Phylogenetic reconstruction was by neighbour joining of MCL-corrected pair-wise distances. F gene sequences rooted with bovine RSV; G gene sequences unrooted. Sequence symbols are colour coded by geography to emphasize the congruence between the phylogenies of the two genome regions. Monophyletic groupings which contain sequences from the 07/08 respiratory season for which sequence data was available from all four referral centres are indicated in shaded boxes. Bootstrap values >70% are indicated.

Phylogenetic analyses comparing the same 58 HRSV isolates sequenced in the F and G gene regions (Fig. 2) reveals congruence between the two datasets with sequence clusters supported by a 70% bootstrap threshold consistent over the two genome regions. (Greater bootstrap support was evident in the G gene, reflective of the greater diversity seen in this region.) Closer inspection of the HRSV-A F and G gene phylogenies revealed two monophyletic lineages (Fig. 2). Directional evolution of the two HRSV-A lineages is evident visually.

### Identification of positively selected sites in the HRSV genes encoding surface proteins

To further understand the evolutionary pressures acting on HRSV and HMPV, analyses to detect codons under positive selection in the F and G genes of both viruses were undertaken. No positively selected codons were identified in the F gene of HRSV clades, findings that contrasted markedly with the 32 positively selected sites detected within the HRSV-A G gene and 5 in the HRSV-B G gene (Table 3). The codon encoding residue 258 was the only residue identified as positively selected for both subtypes.

### Circulating clades of HMPV

**Figure 3:**
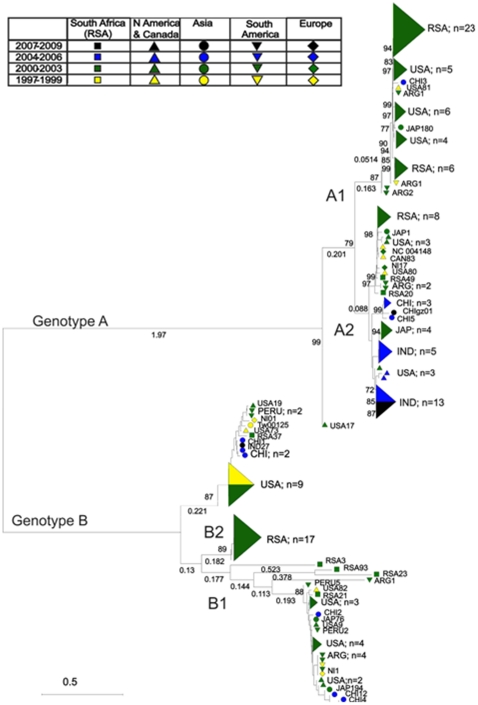
Phylogenetic analysis of HMPV partial G gene sequences (unrooted). Phylogenetic reconstruction was by neighbour joining of MCL-corrected pair-wise distances. Genotypes (A and B) and sub-genotypes (A1, A2, B1 and B2) are indicated. Sequence symbols are colour coded by year of isolation and symbol shape is designated depending on geographic origin of sequence. Bootstrap values >70% are indicated.

HMPV phylogenetic reconstructions revealed 5 main monophyletic groups corresponding with five major clades, one within sub-genotype B1, and two each within sub-genotypes A2 and B2 (labelled 1–5; Fig. 1B). The availability of geographically diverse HMPV-F sequences in Genbank allowed identification of strains from at least two, and up to four continents within clades 2, 3, 4 and 5. Clade 1 was the exception, comprised entirely of strains from Edinburgh and the Netherlands. Older HMPV strains clustered to the internal nodes of the tree. HMPV G protein sequence data available in Genbank spanning 11 years between 1997 and 2008 was analysed phylogenetically (Fig. 3). This also revealed geographically disparate strains interspersed phylogenetically, and that several identified lineages circulated concurrently, confirming the observations drawn from the F gene phylogenetic analysis, and comparable with HRSV. Together with the strong evidence of directional evolution, this is indicative of epidemiologic and evolutionary traits shared by the two members of the *Pneumovirinae*.

HMPV strains from Bangkok were exclusively of genotype A, though previously determined strains of Japanese origin grouped in genotype B (clades 2 and 3). One unusual HMPV genotype A sequence from the Netherlands (NL20850160/08.042) did not group into either sub-genotype A1 or A2. Despite sampling from three globally distributed referral centres collected over two years, we were unable to identify any sequences belonging to the HMPV A1 sub-genotype.

### Positive selection in the HMPV surface proteins

Analysis of the F gene of HMPV-B sub-genotypes yielded only two positively selected codons in the B2 group at residues 391 (*p* = 0.870)) and 400 (*p* = 0.664). Within the G gene of HMPV-A, fourteen codons were positively selected, six were positively selected within HMPV-B1 and 17 sites were identified as under positive selection within HMPV-B2 (Table 3). For HMPV-A, HMPV-B1 and HMPV–B2, sites identified as under positive selection were usually different between groups, except residue 110 which was identified in all three groups.

### HRSV-F and HMPV-F clade turnover

Evolutionary analyses of three HRSV-F and five HMPV-F clades was undertaken to determine the minimum length of time monophyletic lineages of both viruses have been circulating (Table 4). Strict molecular clock models were always used under the assumption that the evolutionary rate within a clade did not vary. Analysis of the HRSV-B clade (clade 1, Fig. 1A) was unable to yield an ESS>200, likely due to the low diversity encompassing most F gene sequences within the subtype and so was excluded from analyses. Indeed, in this clade, visual evidence of directional evolution was not evident from phylogenetic analysis. The *tMRCA* of HRSV-A clades was 17 and 14 years and of HMPV clades was between 11 and 27 years. Genetic diversity across HRSV and HMPV clades was around 2–3% (Table 4). Congruent *tMRCA*, diversity and substitution rates of clades of both viruses (Table 4) support the existence of evolutionary mechanisms common to both virus species.

## Discussion

HRSV and HMPV sequence data generated from isolates collected over 44 years and 26 years respectively were analysed using a variety of techniques, including phylogenetic reconstruction, evolutionary modelling and identification of positively selected sites to gain insight into the epidemiology and evolution of these closely related viruses. Identification of contemporary HRSV and HMPV clades was undertaken for evolutionary modelling, to further understanding of the circulation trends of predominant virus lineages. Evolutionary analyses of the *Pneumovirinae* have not previously been approached in this way.

The evolutionary rates of F gene sequences for HMPV clades in the range of 1.0–1.7×10^−3^ substitutions/site/year (Table 4) are slightly higher, albeit within the 95% highest posterior density (HPD) intervals, than the rates calculated in previous studies sampling across the species of 0.9×10^−3^ [56] and 0.712×10^−3^ substitutions/site/year [57]. It has previously been noted that external branches of the HMPV-F phylogenetic reconstruction have higher dN/dS ratios than internal branches [56]. Taken together, these observations suggest that substitutions are more frequently selected for in the contemporary virus population than previously. This might be explained by an increasing virus population size – random sampling of a larger virus population increases the likelihood of detection of nucleotide changes, and increasing population size increases the probability that residue changes will be selected for.

Previous calculation of the evolutionary rate of HRSV has been undertaken by analysing G gene sequence data, with rates of 1.83×10^−3^ and 1.95×10^−3^ substitutions/site/year determined for subtypes A and B respectively [10], [11]. These rates are slightly higher than those calculated here for the F protein (1.3–1.5×10^−3^ substitutions/site/year). A higher evolutionary rate in the G protein than the F protein has similarly been observed for HMPV [56]. The G protein is under evolutionary pressure due to the host population adaptive immune response to this immunogenic region [45], [63]–[66]. Nucleotide substitutions, most commonly those which are non-synonymous, are frequently selected for, and as nucleotide changes become fixed in the population they are more likely to be captured by the evolutionary analyses, which might explain why the model yields higher rates in this region. Conversely, the extremely low dN/dS ratio and lack of positively selected sites in the F protein [67] provides evidence that changes in the F protein are deleterious, probably due to functional constraints.

For both HMPV and HRSV, we have identified an abundance of sites under positive selection within the G gene, but few within the F gene. In HRSV-A, we identified 32 sites under positive selection. Previous analysis to detect positively selected sites within HRSV-A [10] identified twelve positively selected sites using sequence data spanning a similar time frame, with 48 sequences analysed compared with 139 here; 9 residues were identified by both analyses (111, 117, 215, 226, 262, 274, 276, 290, 297). The previous study used the same program and threshold for significance, and similar models and sampling frame in terms of the number of representative strains and date range analysed. An explanation of this incongruence might be in the differences between the predominantly circulating lineages of the UK and Belgium; these may differentially evolve and/or be under dissimilar structural or immunologic constraints. Alternatively, we may have identified more positively selected sites due to the larger dataset analysed. The HRSV residues identified as under positive selection 142, 206, 274 and 286 have been associated with substitutions in successful antibody escape mutants [68]–[70]. The positively selected residues 215, 217 and 226 fall within a region thought to be immunogenic of neutralising antibodies [71], [72], and residue 297 has previously been identified as a determinant of the integrity of multiple overlapping strain-specific epitopes [73].

An inability of antibodies to select for mutations at sites autonomous to their binding specificity has previously been used to support the notion that HRSV-G epitopes are linear rather than conformational [45]. The greatest distance between any two residues identified as under positive selection within HRSV-A, excluding the conserved region between residues 151–190 (in which we identified one residue under positive selection at position 161), was eight amino acids, which suggests that HRSV-A might potentially generate variability in any epitope of the G protein. The four residues identified as under positive selection in the previous study which were not verified through this work were nevertheless proximal to residues identified as positively selected in this study, with the greatest distance between the two being four amino acids. This supports the notion that within epitopes, changes in any residue might be selected for immune evasion.

For HRSV-B, only five residues were identified as under positive selection, compared with twelve during a previous study. Again, we analysed a greater number of sequences than previous work, though both studies used sequences generated from samples collected over a similar time frame [11]. Here, all the identified sites under positive selection were in the second hyper-variable region in the ectodomain, and only two of the five residues we identified as positively selected were also identified as positively selected previously. Two of the discordant sites we identified were within or downstream of the previously identified [11], [43] 60 nucleotide repeat insertion at the 3′-proximal end of the G protein gene, and so it is possible that these sites were not detected by previous analyses due to limited availability of sequence data for the insert region. Residue 224, the third amino acid not previously identified as such, was determined as positively selected with a probability of *p* = 0.501, and was not detected by the Naïve Empirical Bayes test (used in previous analyses), accounting for the discordancy at this residue between studies.

Six-nucleotide in frame deletions at amino acid positions 159 and 160 reported previously [11] were observed in four of the Newcastle HRSV isolates from 1985, 1986 and 2009. This occurred in different bootstrap-supported lineages of the G region phylogenetic tree, providing strong evidence that this deletion has been independently selected for more than once. Recent identification of two epitopes within the central conserved region of the HRSV G protein ectodomain between residues 151–163 and 164–176 [49] illustrates the immunogenicity of these two peptide regions, while an investigation of the properties of the central conserved domain of HRSV-G showed that the region between amino acids 149–177 played no role in virus infectivity [74]. A loss of these two residues may therefore reduce virus immunogenicity while having no effect on virus infectivity. These observations, together with previous reports of premature stop codons and frame shifts within the subgroup B G protein [71], [73], [75], suggest that HRSV-B may use quite different mechanisms from HRSV-A to evade host immune responses.

A number of residues were identified as positively selected within the G protein of HMPV types A, B1 and B2 (14, 6 and 17) which were differentially located between lineages, in keeping with the observations made of HRSV. There is a predicted cytotoxic T cell epitope between residues 32–41 [76], and within this region one site was identified as positively selected within HMPV-A. The residue identified as under positive selection in all three HMPV-G analyses (residue 105) is not a predicted site of N- or O-glycosylation [51].

Phylogenetic analyses of HRSV and HMPV yielded the common observation that strains isolated from geographically widespread referral centres frequently resolved within the same lineage, reflecting the ability of these viruses to disseminate rapidly on a global scale, and substantiating previous reports of the worldwide distribution of lineages of both viruses [11], [63], [68], [77].

Switching of the predominantly circulating genotype (HMPV)/subtype (HRSV) in a population is widely discussed, but poorly understood in terms of what drives these events or the mechanisms by which they occur. The MRCA estimates for contemporary circulating clades were for HRSV and HMPV 14–17 and 11–28 years respectively, providing conclusive evidence that switching of the predominantly circulating genetic group of both viruses arises independently of novel lineage emergence events. The differences in evolutionary rates between older and more recent HMPV isolates, interpreted here as evidence of an increasing population size, contradicts a previous analysis which showed that one HMPV lineage was increasing in size whereas another was decreasing [57]. Taken together, this information lends to the hypothesis that HMPV (and HRSV) lineages circulate in a cyclic trend of multiple eclipse phases preceding periodic population expansions. Proliferation occurs when the lineage is of minimal susceptibility to the adaptive immune responses of the host population, and a regression in circulating frequency occurs as the host population is increasingly exposed. During the eclipse phase, the virus evolves immune evasive characteristics, which when accumulated sufficiently permit a new phase of widespread circulation.

In summary, we have analysed the molecular epidemiology and evolution of HRSV and HMPV in parallel using the novel approach of clade identification for evolutionary analysis. This work has revealed a number of shared trends, including evidence of both locally and globally circulating lineages of both viruses, significant positive selection acting in the G but not the F genes and a lack of evidence for positive selection being restricted to specific codons. Switching of the predominantly circulating subtype (HRSV) or genotype (HMPV) may be a result of fluctuating circulating frequencies of contemporary clades, which cycle through proliferative and eclipse phases, and is not due to novel lineage emergence events. We suggest that HRSV has the ability to select for residue substitutions at multiple sites within epitopes, contributing to the successful recirculation to high incidence of lineages of this virus.

## Methods

### Sample collection

HRSV and HMPV positive respiratory samples archived between March 2006 and December 2008 at the Specialist Virology Centre (SVC), Royal Infirmary of Edinburgh, UK were identified as described previously [13]. 26 HRSV isolates were collected between 1965 and 2009 from Newcastle, UK [6]. Additionally, nine HRSV and eight HMPV positive samples from Bangkok, Thailand (2006–07 respiratory season) detected as described previously were included in analyses [78] along with 16 HRSV and 16 HMPV variants from the Academic Medical Centre, Amsterdam, Netherlands from the 2007–8 season.

### HRSV and HMPV nucleotide amplification and sequencing in the F and G genes

All HRSV (*n* = 183) and HMPV (*n* = 177) positive respiratory samples were amplified nd sequenced in the 3′ F gene region as previously described [13] (589 and 438 nucleotides respectively). For HRSV, all 26 isolates from Newcastle, seven from Bangkok, 16 from the Netherlands and eight from the UK were amplified in the 3′ G gene region that included both HVRs (780 nucleotides). Combined, the HRSV variants analysed were globally distributed and spanned 44 years. These were analysed alongside available HMPV G gene sequences in Genbank, which encompassed a temporal diversity of 12 years.

HRSV and HMPV RNA was extracted using Qiagen QIAamp viral RNA mini kit and reverse transcribed using Qiagen A3500 reverse transcription system, with extended elongation of 55 minutes and use of random primers. HRSV and HMPV cDNAs were amplified by nested PCR. Reaction mixtures contained 4 µl MgCl2, (25 mM), 0.2 µl dNTP (3 mM), 1 µl each outer primer (10 mM) and 0.08 µl TaqPolymerase. Primers for HRSV-F gene PCR were (outer sense) 916- TAT GGW GTD ATA GAY ACM CCY TGY TGG, (inner sense) 1018- GG RTG GTA YTG TGA YAA TGC AGG, (inner antisense) 1663-CT TAR TGT RAC TGG TGT GYT TYT GGC and (outer antisense) 1682- TWC CAC TYA GTT GRT CYT TRC TTA RTG. HRSV-G gene was amplified by primers (outer sense) 47- CCT GGG AYA CTC TYA ATC AT, (inner sense) 137-TGG CAA TGA TAA TCT CAA C (inner antisense) 117- CCT YTG CTA ACT GCA CT and (outer antisense) 147-GTA TAC CAA CCW GTT CTT A; antisense primers align in the downstream fusion gene. Primers for HMPV F gene amplification were as described previously [13]. 2 µl cDNA was used in the first round and 1 µl first round product was used in the second round reaction. The same cycling conditions were used throughout; 30 cycles at 94°C for 18 s, 50°C for 90 s and 72°C for 30 s, and a terminal 72°C elongation step for 300 s.

Sequences obtained in the course of this study have been submitted to GenBank and assigned accession numbers GU386461–GU386756 (HMPV and HRSV F gene) and HQ731687–HQ731784 (HRSV G gene).

### Phylogenetic analysis

A summary of the computational techniques undertaken for this work and the sequence datasets analysed is tabulated (Table 1). Partial F gene sequences were aligned and genetic distances were calculated using Simmonics v1.9 sequence editor package (www.virus-evolution.org). Phylogenetic trees were constructed from 1000 samplings of maximum composite likelihood (MCL) distances by neighbour-joining method with pair-wise deletions for missing nucleotides in MEGA v4.0. For HRSV, 58 isolates were available for sequencing in both F and G gene regions, and these subsets were phylogenetically analysed separately for comparison using the same methods. HMPV-G sequences downloaded from Genbank were analysed altogether (unrooted). The dataset parameters used for phylogenetic reconstructions are summarized (Table 2).

**Table 1:** Summary of the evolutionary analyses undertaken by taxomonic group.

Taxonomic group	Gene	Analyses undertaken (software used)			
		Nucleotide sequencing (Simmonics)	Phylogenetic (MEGA)	Positive selection (PAML)	Evolutionary (BEAST)
HRSV *spp*	F	**×**	**×**		
HRSV *spp*	G	**×**	**×**		
HRSV-A	F	**×**		**×**	
HRSV-B	F	**×**		**×**	
HRSV-A	G	**×**		**×**	
HRSV-B	G	**×**		**×**	
HRSV clades	F	**×**			×
HMPV *spp*	F	**×**	**×**		
HMPV *spp*	G		**×**		
HMPV-A2	F	**×**		**×**	
HMPV-B1	F	**×**		**×**	
HMPV-B2	F	**×**		**×**	
HMPV-A	G			**×**	
HMPV-B1	G			**×**	
HMPV-B2	G			**×**	
HMPV clades	F	**×**			×

**Table 2:** Sequence datasets for phylogenetic and for positive selection analyses.

Virus group	Gene	Region analysed	Reference strain	Root for phylogenetic reconstruction
HRSV-A	G	Residue 13 to C terminus	Long	HRSV-B
HRSV-B	G	Residue 35 to C terminus	WV/B1/85	HRSV-A
HRSV	F	Residues 358–554	Long	Bovine RSV
HMPV-A1	G	Residue 1 to C terminus	Analysed for positive selection only	
HMPV-B	G	Residue 35 to C terminus	Analysed for positive selection only	
HMPV	G	Residue 35 to C terminus	CAN97-83	Unrooted
HMPV	F	Residues 385–531	CAN97-83	AMPV-C

**Table 3:** Positively selected sites detected in the attachment (G) protein of HRSV and HMPV.

Group	Reference strain	Positively selected sites	Frequency of positively selected sites identified in probability range				
			0.5–0.6	0.6–0.7	0.7–0.8	0.8–0.9	0.9–1.0
**HRSV-A**	Long	101, 104, 106, 111, 115, 117, 121, 122, 123, 126, 127, 131, 142, 146, 161, 206, 215, 217, 226, 230, 233, 250, 258, 262, 274, 276, 280, 286, 289, 290, 291, 297	13	4	5	5	5
**HRSV-B**	WV/B1/85	223, 224, 258, 267/287^1^, 297/317	1	2		1	1
**HMPV-A**	CAN97-83	33, 81, 84, 93, 105, 106, 110, 145, 146, 157, 165, 172, 177, 190	3	2	3	5	1
**HMPV-B1**	CAN97-83	70, 100, 105, 116, 162, 201		2	3		1
**HMPV-B2**	CAN97-83	53, 55, 85, 89, 93, 105, 109, 111, 121, 126, 137, 141, 180, 202, 207, 217, 222	4	8	3	1	1

1 Refers to strains without/with the 60 nt repeated region.

**Table 4:** Substitution rates and estimates of *t_MRCA_* for HRSV and HMPV subtypes and clades, calculated using F gene sequences.

Virus	Range of isolationdates (yrs)	Geneticdist (%)	dN/dS	Evolutionary rate×10^−4^ (95% HPD)	*t_MRCA_*(95% HPD)
HRSV clade 1	2	1.53	0.159	ND^1^	ND^1^
HRSV clade 2	15	2.38	0.0252	13.2 (5.84–20.9)	17 (15–22)
HRSV clade 3	12	2.89	0.0746	15.5 (8.89–22.2)	14 (12–17)
HMPV clade 1	24	2.51	0.104	10.4 (5.50–15.6)	28 (24–33)
HMPV clade 2	11	2.74	0.0746	17.3 (7.14–28.2)	13 (11–16)
HMPV clade 3	6	2.05	0.0212	13.1 (1.19–28.9)	11 (6–22)
HMPV clade 4	26	2.06	0.0705	12.9 (5.75–20.8)	27 (25–30)
HMPV clade 5	16	2.97	0.0229	11.1 (6.38–16.0)	19 (16–24)

1 **Not Determined.**

### Identification of HRSV-F and HMPV-F clades

No systematic method is currently used for identification of distinct HRSV and HMPV lineages. HRSV and HMPV clades (defined as described herein) were identified for the purpose of evolutionary modelling. Phylogenetic analyses of F gene nucleotide sequences of contemporary HRSV and HMPV strains (generated from samples collected since 2007) were constructed as described above for identification of bootstrap-supported monophyletic lineages (values ≥70%) using MEGA v4.0. Subsequent phylogenetic analyses incorporated older monophyletic sequences within the contemporary clades, with visually appropriate limitations of 1.5–3% variation across clades at the nucleotide level and no individual sequence varying from all others within the clade by more than 0.5% at the nucleotide level.

### Identification of positively selected sites in the F protein of HMPV and HRSV clades and the G protein of genotypes/subtypes

Prior to evolutionary analyses, positively selected sites were removed from nucleotide alignments. This was considered necessary as positively selected sites undergo convergent evolution whereas other sites are subject to neutral or nearly neutral drift, and different evolutionary mechanisms inevitably violate assumptions of the SRD06 evolutionary model [59]. Nucleotide alignments of HRSV and HMPV subtypes/sub-genotypes were analysed for positive selection using PAML v4.4. In the attachment protein of HRSV, species level analyses detected all sites in the ectodomain as positively selected due to the high diversity in this region, and similar results were produced for the HMPV species level analyses. This was likely also affected by the large amount of sequence data available. To conserve the maximum diversity in the sequence dataset analysed, analyses for positive selection were undertaken in decreasing increments of taxonomic diversity until satisfactory results were attained. This was found to be the subtype level for HRSV and the sub-genotype level for HMPV. The recommended combination of models 0, 1a and 2a [79] were run and Bayes empirical Bayes results only were considered as recommended. The genome regions analysed for the different virus subgroups are summarized (Table 2). Nucleotide alignments with positively selected sites removed were reanalysed in PAML to confirm no positive selection was detected.

### Clade turnover

Identical sequences (by geography, date and nucleotide sequence) within clades were removed, and evolutionary analyses were restricted to clades with a minimum of 15 non-identical sequences. Three HRSV and five HMPV clades were identified (indicated on Fig. 1). Evolutionary analyses of HRSV and HMPV clades were undertaken using BEAST to calculate evolutionary rates and time since the most recent common ancestor (*tMRCA*) of genetic groups. The F gene region was selected as it was more phylogenetically informative, not subject to positive selection and sequences from a wider geographical and temporal range were available. Clade sequence datasets were run in BEAST using a strict SRD06 model, which allows the third position in a codon to have a different substitution rate to the first and second, until all ESS (expected sample size) values exceeded 200 (recommended). The strict model, as opposed to a relaxed model, assumes that all lineages incorporated within the sequence dataset evolve at the same rate. As the sequence datasets described here were by definition monophyletic groups, the assumption of a nonvariable evolutionary rate within each group was justified. Analyses were run in duplicate to ensure convergence of the posterior distribution, demonstrating repeatability of the result. The coefficient of variation histogram was used to confirm validity of the strict model.
